# Lymphatic muscle cells contribute to dysfunction of the synovial lymphatic system in inflammatory arthritis in mice

**DOI:** 10.1186/s13075-021-02438-6

**Published:** 2021-02-19

**Authors:** Qianqian Liang, Li Zhang, Hao Xu, Jinlong Li, Yan Chen, Edward M. Schwarz, Qi Shi, Yongjun Wang, Lianping Xing

**Affiliations:** 1 Longhua Hospital, Shanghai University of Traditional Chinese Medicine, Shanghai, 200032 China; 2 Key Laboratory of Theory and Therapy of Muscles and Bones, Ministry of Education, Shanghai University of Traditional Chinese Medicine, Shanghai, 200032 China; 3 Department of Pathology and Laboratory Medicine, University of Rochester Medical Center, 601 Elmwood Avenue, Rochester, NY 14642 USA; 4 Center for Musculoskeletal Research, University of Rochester Medical Center, 601 Elmwood Avenue, Rochester, NY 14642 USA; 5 Institute of Spine, Shanghai University of Traditional Chinese Medicine, 725 Wan-Ping South Road, Shanghai, 200032 China

**Keywords:** Inflammatory arthritis, Nitric oxide, Lymphatic muscle cells, TNF, Panax notoginseng saponins

## Abstract

**Background:**

Our previous studies reveal that impaired draining function of the synovial lymphatic vessel (LV) contributes to the pathogenesis of inflammatory arthritis, but the cellular and molecular mechanisms involved are not fully understood.

**Objective:**

To investigate the involvement of lymphatic muscle cells (LMCs) in mediating impaired LV function in inflammatory arthritis.

**Methods:**

TNF transgenic (TNF-Tg) arthritic mice were used. The structure and function of the LVs that drained the hind limbs were examined by whole-mount immunofluorescence staining, electron microscopy, and near-infrared lymphatic imaging. Primary LMCs were treated with TNF, and the changes in proliferation, apoptosis, and functional gene expression were assessed. The roles of the herbal drug, Panax notoginseng saponins (PNS), in arthritis and LVs were studied.

**Results:**

TNF-Tg mice developed ankle arthritis with age, which was associated with abnormalities of LVs: (1) dilated capillary LVs with few branch points, (2) mature LVs with reduced LMC coverage and draining function, and (3) degenerative and apoptotic appearance of LMCs. TNF caused LMC apoptosis, reduced expression of muscle functional genes, and promoted the production of nitric oxide (NO) by lymphatic endothelial cells (LECs). PNS attenuated arthritis, restored LMC coverage and draining function of mature LVs, inhibited TNF-mediated NO expression, and reduced LMC apoptosis.

**Conclusion:**

The impaired draining function of LVs in TNF-Tg mice involves LMC apoptosis. TNF promotes LMC death directly and indirectly via NO production by LECs. PNS attenuates arthritis, improves LVs, and prevents TNF-induced LMC apoptosis by inhibiting NO production of LECs. LMCs contribute to the dysfunction of synovial LVs in inflammatory arthritis.

**Supplementary Information:**

The online version contains supplementary material available at 10.1186/s13075-021-02438-6.

## Introduction

Rheumatoid arthritis (RA) is an autoimmune disorder that typically has symptoms of warm, swollen, and painful joints. It is featured by synovitis with infiltration of inflammatory cells, predominance of catabolic factors including pro-inflammatory cytokines and pro-erosive mediators, and the progressive destruction of cartilage and bone. Clinical study reports that lymph drained from RA joints contains high levels of pro-inflammatory cytokines and chemokines [1], suggesting that the removal of inflammatory factors via the lymphatic vessels (LVs) from synovial space and interstitial tissues may contribute to the pathogenesis of RA. The lymphatic vasculature is composed of capillaries and mature vessels that have different morphologies, structural compositions, and functions. Capillary LVs have a thin layer of lymphatic endothelial cells (LECs) that are stained positively for a lymphatic vessel endothelial hyaluronan receptor Podoplanin or CD31. LECs have closed ends with a button-like structure. When interstitial pressure is higher than that of LVs, these buttons open up and allow interstitial fluid to drain into the capillary LVs. Mature (also called collecting) LVs are covered by 1–2 layers of muscle cells. These lymphatic muscle cells (LMCs) contain both smooth and striated muscle contractile proteins, such as α-smooth muscle actin [2, 3]. Alternating contraction and relaxation of LMCs propel lymph through valves to the draining lymph nodes upstream and eventually to the venous circulation [4, 5].

Using tumor necrosis factor-transgenic TNF-Tg and K/BxN mouse models of chronic inflammatory arthritis, we previously described a synovial lymphatic system in mouse joints. Capillary LVs drain fluid containing catabolic factors and macrophages from synovium and surrounding soft tissues. Mature LVs transport these factors and cells to lymph nodes. The draining function of the synovial lymphatic system can be monitored by numerous imaging modalities that we have developed, such as near-infrared lymphatic imaging to measure pluses/contraction and tracer clearance of mature LVs efferent joints [6, 7]. Our findings demonstrated that the synovial lymphatic system is closely related to the progression and flare of arthritic joints, and its draining function is impaired in RA joints [8]. However, the cellular and molecular mechanisms responsible for impaired lymphatic function in RA are not yet fully understood.

Nitric oxide (NO) is a free radical gas transmitter that regulates various biological functions in the body. NO and its inducer, inducible nitric oxide synthase (iNOS), play an important role in inflammatory arthritis. NO levels are high in the serum, urine, and synovial fluid of RA patients [9, 10]. iNOS is undetectable in healthy joints but markedly increased in RA synovium [11–14]. NO regulates LV contraction using a vasodilatory signal in LMCs. We reported that TNF promotes LECs to produce a large amount of NO, which inhibits muscle functional gene expression by LMCs. The herbal medicine, formic acid, prevents TNF-induced LEC NO, restores LV contractions, and attenuates arthritis in TNF-Tg mice [15]. This study shows that abnormal LECs contribute to lymphatic dysfunction in RA. However, the involvement of LMCs in this process has not been investigated. Because both TNF and high levels of NO cause cell apoptosis [16, 17], we hypothesize that TNF may have a similar pro-apoptotic effect on LMCs.

Panax notoginseng (Burk.) is a herbal medicine used to treat patients with cardio- and cerebrovascular diseases, inflammation, and injury-caused internal and external bleeding [18]. Total saponins of Panax notoginseng (PNS) are the biologically active constituents of Panax notoginseng [19, 20]. PNS extract inhibits LPS-induced macrophage activation [21] and intracellular free calcium concentration in neutrophils [22]. It also reduces levels of NO and iNOS in lung tissue [23]. We found that PNS promotes lymphangiogenesis in a zebrafish screening system [24]. However, if PNS affects LVs in RA has not been studied.

In the current study, we used TNF-Tg mice, a mouse model of inflammatory arthritis, to study the structural changes of LVs, LECs, and LMCs. We focused on how TNF affects LMC function and if PNS could prevent TNF-mediated LMC inhibition and restore the structural integrity of LVs in RA.

## Materials and method

### Animals

TNF transgenic (TNF-Tg line 3647) mice were originally obtained from Dr. G. Kollias and back-crossed with C57BL/6 mice for more than 10 generations. This line of TNF-Tg mice carries one copy of the human TNF transgene and develops chronic arthritis relatively slowly. TNF-Tg mice have healthy ankle joints when they are 1 month old. When TNF-Tg mice are 2.5 months old, they develop mild ankle joint inflammation and bone erosion, which become more severe after 5 months. In this study, all mice were housed as 5 mice per cage in specific pathogen-free (SPF) rooms. To examine the effect of the herbal drug total saponins of Panax notoginseng (PNS, National Institutes for Food and Drug Control, CAS No: 88,105-29-7, Lot No: 110,870-201002, purity > 98.5) on arthritis and lymphatic draining function, 2.5-month-old TNF-Tg mice (*n* = 9) were randomized to (1) PNS and (2) saline control groups. PNS (80 mg/kg) or saline was given by gavage once a day for 12 weeks based on mouse body weight. WT littermates (*n* = 8) were treated with saline as the negative control.

The sample size was calculated based on the inflammation area data from our previous study [15] using the PASS11 software (2011 NCSS, LLC). We used one-way analysis of variance power analysis. The power (1-Beta) is 0.8, alpha (significance level) is 0.05, *k* (number of groups) is 3, group allocation ratios are equal, hypothesized means are 0.00, 64.67, and 16.5, while *s* (standard deviation of subjects) is 6.8. The numeric result is *n* = 2/group. To avoid insufficient sample size caused by death by gavage, we used more than 4 mice/group.

At the end of the experiment, mice were subjected to NIR-ICG imaging for lymphatic draining function. Ankle joints were harvested for histology. In the second experiment, all the mice were generated and kept in the SPF room of Shanghai Model Organisms Center and were housed in 5 mice per cage with a 12-h light/dark cycle and 25 °C room temperature with free access to food and water. Two-month-old Sprague-Dawley rats purchased from Shanghai Laboratory Animal Inc. were used for isolating LMCs as we described previously [15]. All animal procedures were followed by the Guiding Principles for the Care and Use of Laboratory Animals approved by the Animal Regulations of National Science and Technology Committee of China and the Animal Care and Use Committee at the University of Rochester.

### Near-infrared indocyanine green (NIR-ICG) lymphatic imaging

NIR-ICG lymphatic imaging was performed according to the method that we described [25–29]. In brief, fur was removed from the legs with hair removal lotion. In the case of respiratory anesthesia, the mice were fixed on the thermostat in the prone position, and an ICG solution (0.1 mg/ml, 6 μl) was injected intradermally into the footpads. The dynamics of ICG fluorescence over the entire leg was visualized under an infrared laser to observe the collecting lymphatic vessels efferent foot area. The ICG fluorescence in the region of interest (ROI) over the footpad and leg was immediately recorded for 30–60 min. Sequential images were analyzed for ICG intensity using the ImageJ software (ImageJ bundled with 64-bit Java 1.8.0_112, https://imagej.nih.gov/ij/download.html), resulting in an outcome measure of LV contraction, which represents the ICG pulses that pass the ROI within 100 s, as we described previously [7]. The imaging analysis was done by JLL, who was blinded to the group’s allocations.

### Histology

The ankle joints were fixed in 10% phosphate-buffered formalin, decalcified in 10% EDTA, and embedded in paraffin. A series of sections (4 μm) were cut. A total of 10 sections were collected and divided into 3 levels. Each level was 40 μm from the previous level. One section from each level was stained with hematoxylin and eosin (H&E). The inflammatory area and bone area were measured by histomorphometry. The data were presented as the mean from 3 levels cut from each joint sample. Samples were analyzed by YC who was blinded to the group’s allocations.

### Immunofluorescence staining

Antibodies included rabbit anti-mouse lymphatic vessel endothelial hyaluronan receptor (LYVE-1, Abcam Inc., Cambridge, MA, #ab14917), hamster anti-mouse Podoplanin (PDPN, Abcam Inc., #ab11936), PE-conjugated rat anti-mouse CD31 (BD Pharmingen, MD, #553373), FITC-conjugated mouse monoclonal anti-mouse α-smooth muscle actin (SMA, Sigma-Aldrich Corp., Saint Louis, MO, #F3777), Alexa 488-conjugated goat against rabbit secondary antibody (Molecular Probes, Eugene, #A11001), and Alexa-546 goat anti-hamster IgG (H+L) (Molecular Probes, #A211).

For whole-mount staining of LVs, fur was removed with hair removal lotion, and ankle tissues (Supplemental Fig. 1) were fixed in 10% formalin and blocked with 3% milk in 0.3% Triton X-100. Tissues were incubated with anti-LYVE-1 (1:1000) and followed by Alexa 488-conjugated secondary antibody (1:400) for LYVE-1^+^ lymphatic capillaries; (2) FITC-anti-αSMA (1:400) and PE-anti-CD31 (1:80) or anti-PDPN (1:1000) for mature LVs, as have been reported [2, 3, 30–33]. Tissues were then mounted with glycerin and imaged with a fluorescence microscope (Olympus IX 71).

For immune-staining of LMCS, cells were fixed with 10% formalin, blocked with 0.2% Triton-100 in 1% BSA, and stained with FITC-anti-αSMA (1:400). Cells were observed under a fluorescence microscope (Olympus IX 71).

### Quantification of lymphatic vessels

We measured the diameter and number of branch points to assess lymphatic capillaries and the percentage of αSMA+LMC coverage to evaluate mature LVs, commonly used outcome measures in lymphatic studies using whole-mount staining [30, 34]. To qualify capillary LVs, LYVE-1-stained whole-mount samples that contain 22–30 vessels were imaged at low magnification (× 4). The diameters of LVs in the entire sample were measured by outlining a vessel manually and calculating the distance between both sides of the vessel using the Image-Pro Plus 5.0 software (Media Cybernetics, Inc., Bethesda, MD). The number of branch points was measured on the same image by outlining a region of interest (ROI) at the same location and counting all branch points in the ROI. For the quantification of the percentage of αSMA coverage on mature LVs, CD31/αSMA or PDPN/αSMA double positive-stained vessels were imaged at high magnification (× 10). Four images in the different areas were taken from each sample. The percentage of αSMA coverage was calculated by the following equation: αSMA+ area/CD31+ or PDPN+ area at LVs × 100%. The data of 4–5 images were combined. Data were analyzed by HX, who was blinded to the group’s allocations.

### Transmission electron microscopy

Collecting lymphatic vessels efferent foot area were isolated and fixed with 2.5% glutaraldehyde/4% formaldehyde in 0.1 M sodium cacodylate buffer. The specimens were post-fixed in 1.0% buffered osmium tetroxide, dehydrated through a graded series of ethanol, infiltrated/embedded into EPON/Araldite epoxy resin, and polymerized at 60 °C for 2 days. One-micron-thick sections were cut and stained with Toluidine Blue to identify the lymphatic vessel. Seventy-nanometer-thin sections were then cut and mounted onto 200 mesh carbon-coated nickel grids and were stained with uranyl acetate and lead citrate for ultrastructural examination. The grids were examined and photographed using a Hitachi 7650 transmission electron microscope with an attached Gatan 11 megapixel Erlangshen digital camera.

### Isolation of LMCs

We followed a published protocol [35]. Mesenteric lymphatic vessels from 2-month-old rats were identified by injecting 10 μl 0.5% Evans blue into the mesenteric lymph nodes. Blue-stained lymphatic vessels that were easily separated from the blood vessels were harvested, cut into small pieces, and transferred to a 1% gelatin-coated plastic tissue culture dish. Cells were cultured in high-glucose Dulbecco’s modified Eagle’s medium supplemented with 20% FBS, 2 mM sodium pyruvate, 2 mM l-glutamine, and antibiotics; the culture medium was changed every 3 days. Muscle cells covering LVs migrated from the vessels after 3–4 days, and vessel segments were removed aseptically. The cells were trypsinized after 7–10 days with 0.25% trypsin in 0.02% EDTA and transferred to a new gel-coated dish (passage 0). After 1–2 weeks, the cells reached confluence and were split into two dishes (passage 1). Cells from passages 3–6 were used.

### Cell growth and apoptosis assays

Cell growth was examined by an MTT assay (Sigma, # CGD1) according to the manufacturer’s instructions. In brief, cells were seeded at a density of 2.4 × 10^3^cells/well in 96-well plates in triplicate. After treatment with 10 ng/ml TNFα (R&D, Minneapolis, #410) for different times, cells were incubated with 20 μl of MTT solution at 37 °C for 4 h, followed by 200 μl of MTT solvent to terminate the reaction. The plates were read at 570 nm using a benchmark microplate reader (Bio-Rad). Cell apoptosis was assessed by an Annexin V FITC kit (BD Biosciences, #556570) according to the manufacturer’s instructions. The analysis was conducted on a FACS Calibur flow cytometer using the FlowJo 7.6 software. Cells were also treated with 0, 2.2, 6, and 20 ng/ml IL-6 (SinoBiological, #80076-RNAE) for 1–7 days, and cell growth and apoptosis were examined.

### Co-culture of LECs and LMCs

A murine LEC cell line established from Freund’s adjuvant-induced benign lymphangiomas was provided by Dr. S. Ran from the University of Illinois [36]. We have used this LEC cell line in previous studies [15, 37]. Cells were seeded on the upper chamber at 3 × 10^5^cells/well of a transwell insert and were treated with 10 ng/ml TNF +/− Ami or PNS for 24 h. The transwell inserts with LECs were then transferred into a 6-well plate, which had already been coated with 2 × 10^5^ LMCs. After 5 days of co-culture, the upper chamber with LECs was removed, and LMCs were harvested for further analysis.

### Nitric oxide (NO) levels

LECs were seeded at 10^6^/well in six-well plates overnight and pre-treated with 1 mM aminoguanidine hemisulfate salt (Ami), a selective iNOS inhibitor (Sigma, #M7033) or PNS (100 μg/l) for 3 h and then with 10 ng/mL TNF for 24 h. Supernatants were collected, and nitrite levels assessed using a NO assay kit (Nanjing Jian Cheng Bioengineering Institute, Nanjing, China, #A012).

### Real-time PCR

The total RNA was isolated using TRIzol Reagent (Invitrogen, Carlsbad, CA), and Complimentary DNA was prepared from the total RNA using GeneAmp RNA PCR core kit (Applied Biosystems, Foster City, CA). Quantitative PCR amplification was performed in triplicate assays with gene-specific primers and iQ SYBR Green Supermix (both from Bio-Rad Laboratories, Hercules, CA) in an iCycler real-time PCR machine, according to the manufacturer’s instructions. The relative abundance of each gene was calculated by subtracting the CT value of each sample for an individual gene from the corresponding CT value of *β-actin* (ΔCT). ΔΔCT was obtained by subtracting the ΔCT of the reference point. These values were then raised to the power two (2^ΔΔCT^) to yield the fold expression relative to the reference point. Expression levels of muscle functional genes were examined using sequence-specific primers (Table 1).

**Table 1:** Sequences of primers used in the real-time polymerase chain reaction

Genes	Sequences of primers	GenBank accession number	Annealing Tm(°C)	Product size (bp)
SM α2-actin	F: 5′ TATTCTGTCTGGATCGGCGG 3′ R: 5′ ACATTCACAGTTGTGTGCTAGAG 3′	NM_031004.2	60	196
SM α1-actin	F: 5′ CATGGACCATTATGATTCCCAGC 3′ R: 5′ CTTTGCGCAGGTGGGAGTTG 3′	NM_031005.3	57	143
SM α22-actin	F: 5′ TCTCCTTCCAGCCCACAAAC3′ R: 5′ TTCACGGCTCATGCCATAGG3′	NM_031549.2	60	82
h1-calponin	F: 5′ TGGCCCAGAAATACGACCAC3′ R: 5′ CCGGCTGGAGCTTGTTGATA3′	NM_031747.1	60	144
Nfkb2	F: 5′ TCTGGTCACCAAGCTCCATGCTAA 3′ R: 5′ TGGATGTCAGCACCAGCCTTTAGA 3′	NM_001008349.1	60	129
Smooth muscle myosin, heavy chain 11	F: 5′ TCCGGTGTTCTCCTGCTAGT 3′ R: 5′ GGGCCATTGGGCTGTTTATG 3′	NM_001170600.1	60	134
β-Actin	F: 5′ TTGCTGACAGGATGCAGAAGGAGA 3′ R: 5′ ACTCCTGCTTGCTGATCCACATCT 3′	NM_031144.3	60	159

### Western blot analysis

Whole-cell lysates were harvested, and samples (30 μg protein/lane) were fractionated by SDS-PAGE and transferred to the nitrocellulose membranes. Immunoblotting was carried out using antibodies to smooth muscle myosin heavy chain 2 (1:1000, Abcam Inc., #ab53219), h1-Calponin (1:1000, Abcam Inc., #ab46794), and β-actin (1:5000, Sigma, #A2228). Bands were visualized using ECL chemiluminescence (Amersham).

### Flow cytometry

LMCs (1 × 10^5^) were cultured on a 6-cm cell culture dish overnight and were treated with TNFα (10 ng/ml) for 0.5, 1, 2, or 10 h. Cells were harvested by trypsin digestion and were fixed with a Fixation and Permeabilization Solution (BD Biosciences, #554722) according to the manufacturer’s instructions. Cells were stained with the rabbit antibody against pERK1/2 (Cell Signal Tech, #4695) followed by the anti-rabbit Alexa 488 antibody (Cell Signal Tech, #4412S). Flow cytometry (BD, LSR Fortessa SORP) was performed to detect samples, and data were analyzed using the software FlowJo (BD, V10).

### Statistical analysis

Data are presented as means ± SD. Statistical analyses were performed with the SPSS 16.0 software. The Student *t* test was used for the differences between 2 groups after the *F* test for homogeneity of variance. One-way ANOVA test followed by Dunnett’s multiple comparison test. The analysis of variance in repeated measurement design followed LSD-*t* post hoc test was used for repeated measurement data comparisons. Differences were considered statistically significant when *p <* 0.05.

## Results

### Abnormal lymphatic vessel structure is associated with the progression of arthritis in TNF-Tg mice

**Fig. 1:**
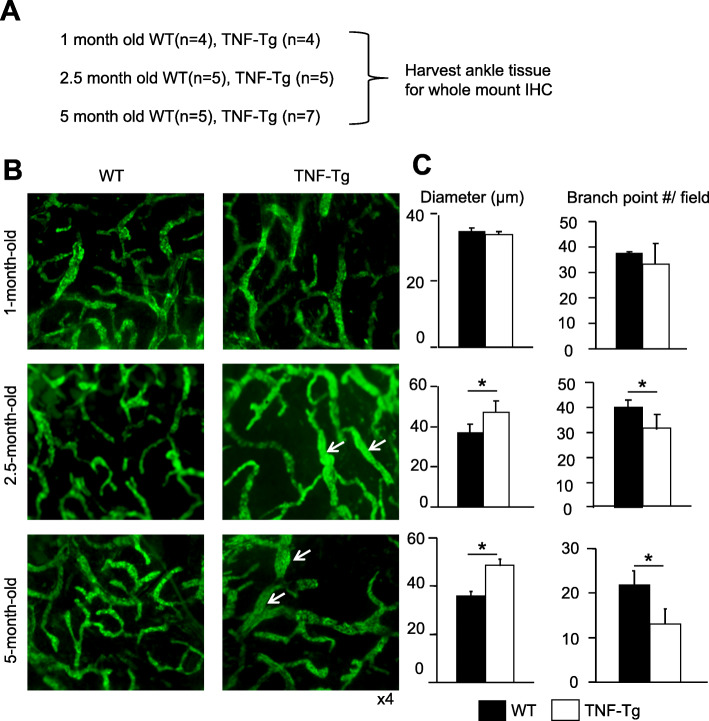
Increased diameters and reduced branch points of capillary lymphatic vessels in TNF-Tg mice. **a** Schematic illustration shows the experimental design; ankle tissues from 1-, 2.5-, and 5-month-old TNF-Tg mice and WT littermates were subjected to whole-mount immunofluorescence staining with an LYVE-1 antibody to identify capillary LVs. **b** Representative images show dilated LVs in 2.5- and 5-month old TNF-Tg mice (arrows). **c** Quantification of diameters and branch points. Values are mean ± SD. *n* = 8–9 legs from 4 to 7 mice per group. *p < 0.05 by the Student *t* test

**Fig. 2:**
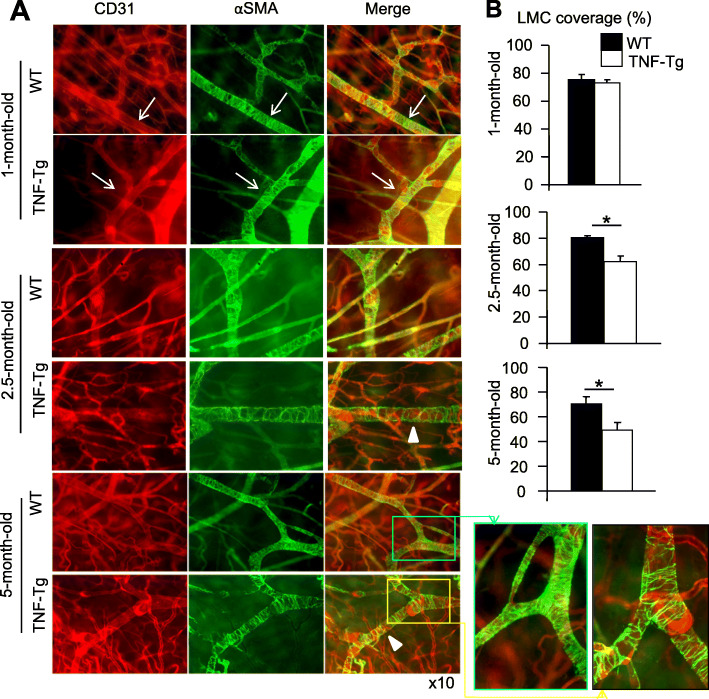
Reduced lymphatic muscle cell coverage in mature lymphatic vessels that drain arthritic joints of TNF-Tg mice. Ankle tissues from Fig. 1 were subjected to whole-mount double immunofluorescence staining with CD31 (red) and α-smooth muscle actin (αSMA, green) antibodies to identify CD31 and αSMA double-positive mature LVs (indicated by white arrows). **a** Representative images (magnification × 10) show reduced vessels that are covered by αSMA+ LMCs in 2.5- and 5-month-old TNF-Tg mice. **b** Quantification of the percentage of vessels that are covered by αSMA+ LMCs. Values are mean ± SD. *n* = 8–9 legs from 4 to 7 mice per group. *p < 0.05 by the Student *t* test

To examine potential alternation of the LV morphology during the progression of arthritis, we established a whole-mount immunofluorescence staining protocol that enables us to visualize global changes of LVs, including lymphatic capillaries and mature vessels, at the mouse ankle where arthritis occurs in TNF-Tg mice. We used an anti-LYVE-1 antibody to identify LYVE-1+ capillary LVs (Fig. 1) and anti-CD31 and αSMA antibodies to detect CD31+/αSMA+ mature LVs (Fig. 2) according to the literature [30] and our recent work [34]. We compared results from 1- (*n* = 4), 2.5- (*n* = 5), and 5-month-old (*n* = 7) TNF-Tg mice and their WT littermates (*n* = 4 for 1-, 5 for 2.5-, and 5 for 5-month-old mice) (Fig. 1a) when 1-month-old TNF-Tg mice have normal joints, 2.5-month-old have mild RA, and 5-month-old have severe joint inflammation and bone erosion. Abnormal LVs were observed starting in 2.5-month-old TNF-Tg mice, which became worse in 5-month-old mice. LYVE-1+ capillary LVs had increased diameter and decreased branch points (Fig. 1b, c), and mature LVs showed significantly reduced LMC coverage (Fig. 2a, b) compared to those of WT littermates, suggesting more immature LVs in TNF-Tg joints because LMC coverage is a marker of vessel maturation [35, 38, 39].

### Ultra-structural changes of endothelium and muscle cells in lymphatic vessels drain arthritic joints of TNF-Tg mice

**Fig. 3:**
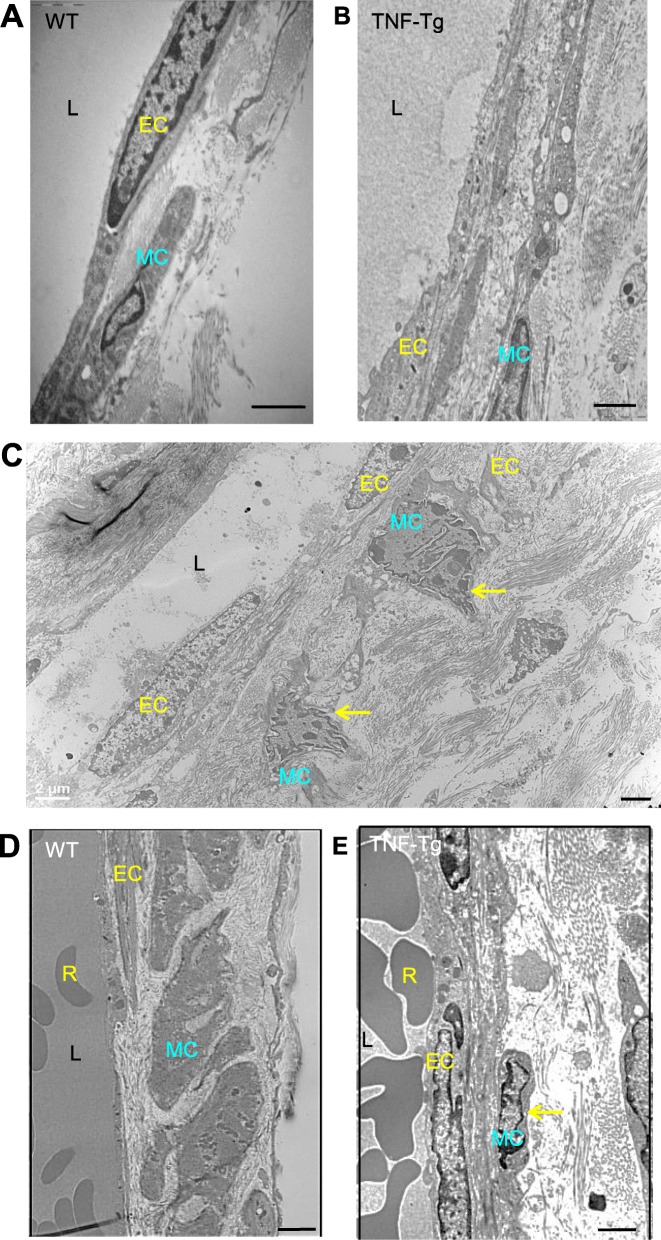
The degenerative and apoptotic appearance of lymphatic muscle cells on the lymphatic vessels that drain arthritic joints of TNF-Tg mice with severe arthritis. Collecting LVs efferent foot area and adjacent blood vessels from 5- to 11-month-old TNF-Tg mice and WT littermates were subjected to electron microscopy. *n* = 3 mice/group. Representative images are shown. **a** An LV wall from a WT mouse, containing a lymphatic endothelial cell, lymphatic muscle cell, and empty lumen. **b**, **c** An LV wall from a TNF-Tg mouse showing contracted and smaller lymphatic muscle cells with condensed nuclear chromosome or apoptotic appearance (arrows). **d** A blood vessel wall from a WT mouse containing a blood endothelial cell, smooth muscle cells, and red blood cells in the lumen. **e** A blood vessel wall from a TNF-Tg mouse showing a smaller smooth muscle cell with nucleus condensation (arrow). EC, endothelial cell; MC, muscle cell; L, lumen; R, red blood cell. Bars are 2 μm

We performed electronic microscopy on LVs that drain the arthritic joints of TNF-Tg mice. Ultra-structural examination revealed that the WT mouse LV had a regular endothelial cell layer, a flat lymphatic muscle cell, and an empty lumen (Fig. 3a). In contrast, there was a noticeable change in the morphology of LVs from TNF-Tg mice (Fig. 3b, c). The endothelium had vacuoles and obvious intraluminal protrusion. More importantly, the smooth muscle cells were degenerated with cytoplasm and condensed nuclear chromosomes, an indicator of cell apoptosis. Similar morphologic changes were also observed in the blood vessels. WT mouse blood vessels were filled with blood cells in the lumen and had regular endothelial cell wall and fat smooth muscle cells (Fig. 3d). However, the TNF-Tg mouse blood vessels were shrinking, and the smooth muscle cells had nucleus condensation (Fig. 3e). Thus, muscle cells covering both lymphatic and blood vessels of TNF-Tg mice have degenerative and apoptotic appearance.

### TNF inhibits the survival and functional gene expression of lymphatic muscle cells

**Fig. 4:**
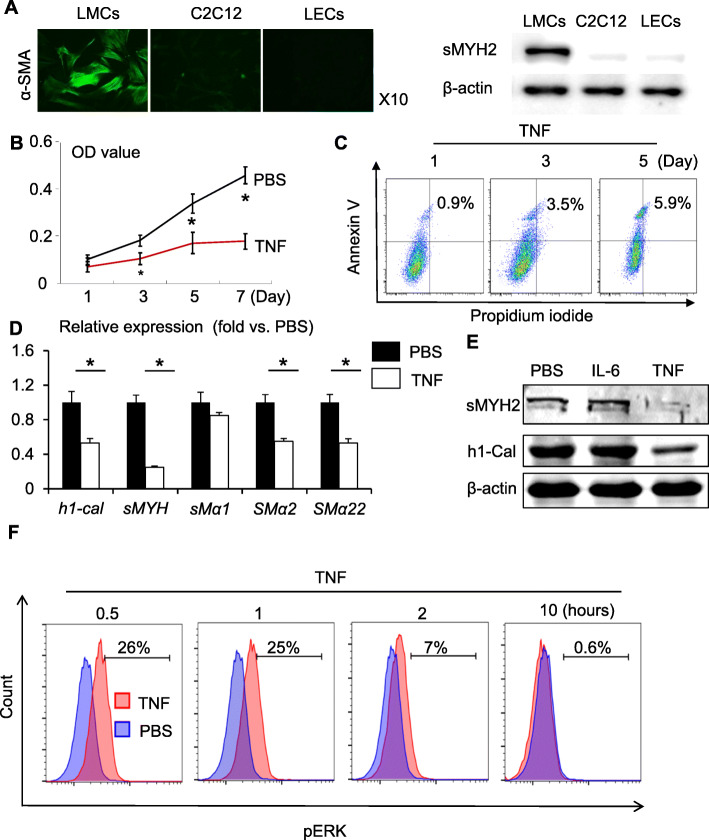
TNF inhibits survival and muscle functional gene expression of lymphatic muscle cells. **a** LMCs were isolated from rat mesenteries. Expression of genes specific for smooth muscle cells, α smooth muscle actin and smooth muscle myosin heavy chain 2 (sMYH2), was determined by immunostaining and Western blot analysis, respectively. C2C12 myoblast precursor cells and LECs were included as negative controls. **b** LMCs were treated with 10 ng/ml TNF. Cell growth was assessed by an MTT assay. Values are mean ± SD of 3 samples. *p < 0.05 by analysis of variance in repeated measurement design followed the LSD-*t* post hoc test. **c** Cell apoptosis was determined by flow cytometry. **d** LMCs were treated with TNF for 24 h. Expression levels of muscle functional genes were determined by qPCR. The fold changes were calculated using PBS-treated cells as 1. Values are mean ± SD of 3 samples. *p < 0.05 by the Student *t* test. **e** LMCs were treated with 20 ng/ml TNF and 20 ng/ml IL-6 for 24 h. The protein levels of sMYH2 and h1-Calponin were determined by Western blot analysis. **f** LMCs were treated with 10 ng/ml TNF for various time points. The phosphorylation of ERK1/2 was examined by flow cytometry. Experiments were repeated 3 times with similar results

Little is known about the involvement of LMCs in the pathogenesis of arthritis. We established a protocol to culture primary LMCs and confirmed that these cells expressed specific smooth muscle proteins: α smooth muscle actin and smooth muscle myosin heavy chain 2 (sMYH2). As negative controls, C2C12 cells, a mesenchymal and muscle progenitor line, and LECs did not express these proteins (Fig. 4a). We examined the morphology and expression of smooth muscle proteins in LMCs after several passages. Cells at the early passages were flat and large and expressed high levels of sMYH2. With increased passaging, the cells became smaller and lost sMYH2 expression (Supplemental Fig. 2). Thus, we used LMCs from the 3rd to 6th passage in our study. We treated LMCs with TNF and found that TNF significantly inhibited cell growth (Fig. 4b), increased the percentage of apoptosis (Fig. 4c), and reduced the expression of muscle functional genes, including h1-calponin, smooth muscle myosin heavy chain 11, smooth muscle actin 2, and smooth muscle actin 22 (Fig. 4d). TNF markedly decreased the expression of sMYH2 and h1-calponin proteins (Fig. 4e) and promoted the phosphorylation of ERK in LMCs (Fig. 4f). To investigate if cytokines other than TNF also affect LMC function, we treated cells with IL-6, an important cytokine for arthritis. IL-6 did not affect the growth and apoptosis (Supplemental Fig. 3), or the expression of sMYH2 and h1-calponin proteins (Fig. 4e)*.* These data indicate that TNF inhibits the survival and functional gene expression of LMCs.

### Total saponins of Panax notoginseng attenuates arthritis, restores structural integrity, and improves the draining function of lymphatic vessels in TNF-Tg mice

**Fig. 5:**
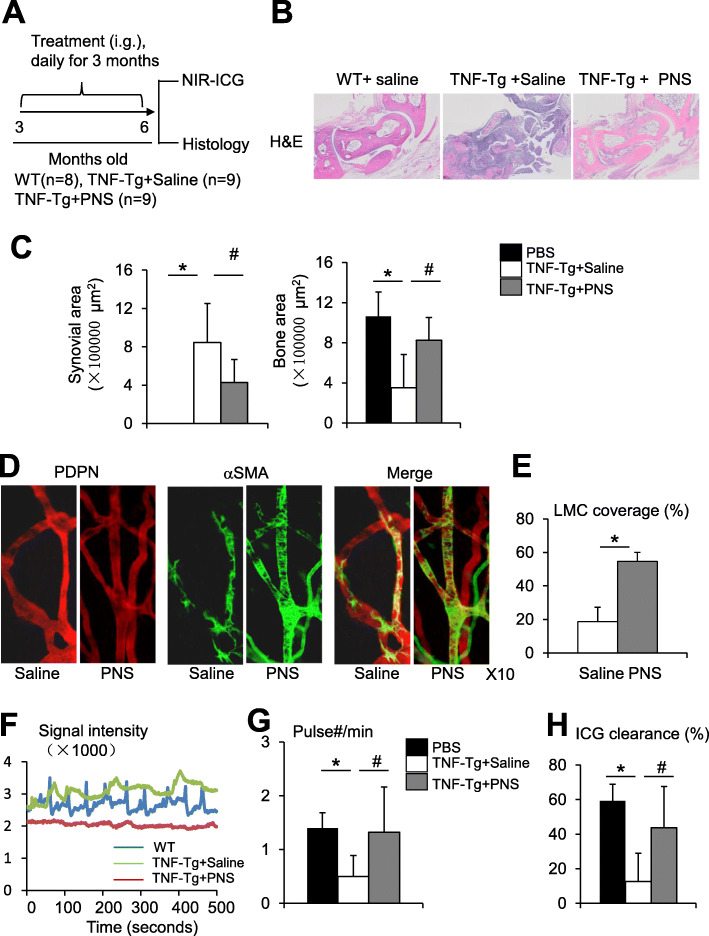
PNS attenuates arthritis, restores structural integrity, and improves the draining function of lymphatic vessels in TNF-Tg mice. **a** Schematic illustration shows the experimental design, 3-month-old TNF-Tg mice were treated with PNS or saline by gavage daily for 3 months. WT littermates were treated with saline as a negative control. Mice were subjected to NIR-ICG lymphatic imaging before being sacrificed. **b** Representative images of H&E- and TRAP-stained sections show decreased inflammation, bone loss, and osteoclasts in PNS-treated mice. **c** Inflammatory area, bone area, and TRAP+ area. Values are the mean ± SD of 5–6 mice. *p < 0.05 vs. WT mice, ^#^*p* < 0.05 vs. PNS-treated TNF-Tg mice by one-way analysis of variance followed by Dunnet post hoc test. **d** Representative images of whole-mount-stained sections show PDPD (red) and αSMA (green) double-positive mature LVs. **e** The percentage of vessels that are covered by SMCs. Values are mean ± SD of 6 mice *p < 0.05 by the Student *t* test. **f** Representative NIR-ICG images show a marked difference in the frequency of pulses among vehicle- and PNS-treated TNF-Tg mice. **g** Lymphatic pulses. Values are mean ± SD of 12–18 legs. *n* = 8–9 mice/group by one-way analysis of variance followed by the Dunnet post hoc test. **h** ICG clearance. Values are mean ± SD of 16–18 legs. *n* = 8–9 mice/group. *p < 0.05 vs. saline-WT, ^#^*p* < 0.05 vs. saline-TNF-Tg mice by one-way analysis of variance followed by the Dunnet post hoc test

Total saponins of Panax notoginseng (PNS) is an herbal drug that has been used to treat patients with arthritis. It has anti-inflammatory effects on macrophages [21]. We found recently that PNS promotes lymphangiogenesis using a zebrafish screen system [24]. We hypothesize that PNS may affect LVs as a new mechanism of action to treat arthritis. We treated TNF-Tg mice (*n* = 9/group) orally with PNS (80 mg/kg) or a saline vehicle once a day for 3 months and examined the joint pathology by histology (Fig. 5a), LV structure by whole-mount staining, and LV draining function by NIR-ICG imaging. Eight WT littermates of the same age were used as a negative control. PNS showed no significant effect on the weight of TNF-Tg mice (Supplemental Fig. 4) and did not show any adverse events. Histologic examination of H&E-stained sections demonstrated severe synovial inflammation and bone erosion in TNF-Tg mice, which were significantly reduced by PNS treatment (Fig. 5b, c). Whole-mount staining indicated that PNS significantly increased LMC coverage of LVs in TNF-Tg mice (Fig. 5d, e). NIR-ICG imaging revealed the absence of lymphatic pulses and reduced ICG clearance in vehicle-treated TNF-Tg mice, as we previously reported [6, 7, 28, 40, 41]. Both were improved by PNS treatment (Fig. 5f–h). These data indicate that apart from the reduction of joint tissue damage, PNS restored the structural integrity of the LVs and improved LV draining function in TNF-Tg mice.

### PNS blocks the inhibitory effects of TNF on lymphatic muscle cells via lymphatic endothelial cells

**Fig. 6:**
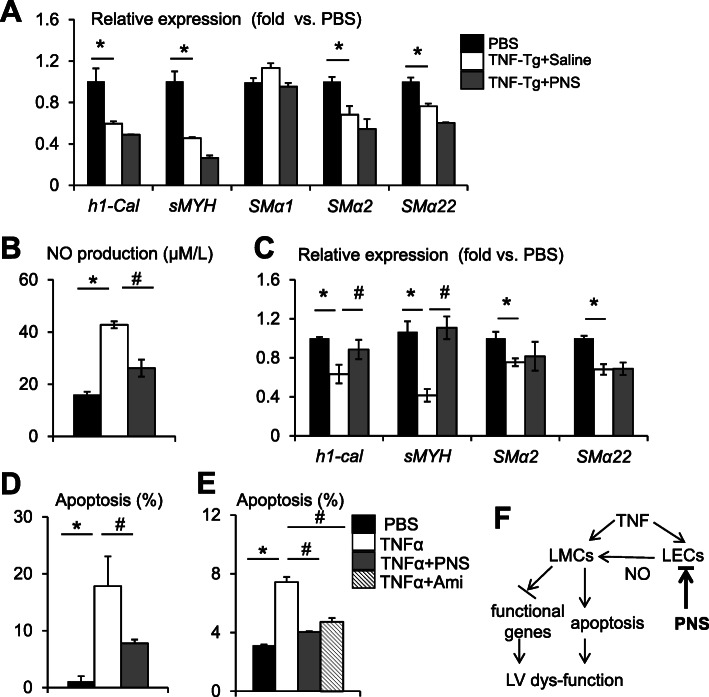
PNS blocks the inhibitory effects of TNF on lymphatic smooth muscle cells via lymphatic endothelial cells. **a** LMCs were treated with PBS or 10 ng/ml TNF ± 100 ng/ml PNS for 24 h. The expression of muscle functional genes was examined by qPCR. The fold changes were calculated using PBS-treated cells as 1. Values are mean ± SD of 3 samples. **b** LECs were treated with PBS or TNF ± PNS for 24 h. NO levels in the conditioned medium were assessed by the Greiss method. Values are mean ± SD of 3 samples. **c** LECs were pre-treated with PBS or TNF ± PNS for 24 h and were then co-cultured with LMCs for an additional 24 h. The expression of muscle functional genes in LMCs was determined as in **a**. **d** LMCs were treated with PBS or 10 ng/ml TNF ± 100 ng/ml PNS for 24 h. Annexin V+/propidium iodide+ apoptotic cells (%) were examined by flow cytometry, as in Fig. 4. **e** LECs were pre-treated with PBS or TNF ± PNS or 1 mM Ami for 24 h and were then co-cultured with LMCs. Apoptotic cells (%) were determined as in **b**. Values are mean ± SD of 3 samples. Three experimental repeats. *p < 0.05 vs. PBS-treated, ^#^*p* < 0.05 vs. PNS-treated cells by one-way analysis of variance followed by the Dunnet post hoc test. **f** A working model showing how PNS affects LMCs in the presence of TNF

To investigate the potential mechanisms that mediate the effect of PNS on LVs, we treated LMCs with PNS in the presence of TNF to determine if PNS could prevent TNF-mediated down-expression of muscle functional genes in these cells. PNS had no effect on TNF-mediated downregulation of *hi-Cal*, *sMYH*, *SMa2*, and *SMa22* (Fig. 6a), suggesting that PNS unlikely affects LMCs directly. We reported previously that TNF promotes LECs to produce NO, leading to the inhibition of muscle functional gene expression by LMCs in the LEC-LMC co-culture system [15]. Interestingly, PNS significantly reduced TNF-induced NO production by LECs (Fig. 6b) and restored the functional gene expression by LMCs that were co-cultured with TNF-pre-treated LECs (Fig. 6c). In addition, PNS partially reduced TNF-induced LMC apoptosis when it was directly added to the LMC culture (Fig. 6d). However, PNS was completely prevented TNF-induced LMC death if it was added to LECs that were pre-treated with TNF. The use of the specific iNOS inhibitor Ami had the same result (Fig. 6e). These data suggest that PNS blocks the inhibitory effects of TNF on LMCs mainly via LECs, and that this process may involve NO-mediated mechanisms (Fig. 6f).

## Discussion

Our previous studies using TNF-Tg mice, a mouse model of rheumatoid arthritis (RA), demonstrated that inhibition of lymphangiogenesis via VEGFR-3 blockade increases [26], while stimulation of lymphangiogenesis via VEGF-C administration decreases the severity of joint inflammation and tissue damage [41]. These studies indicate that sufficient lymphatic drainage is beneficial for RA and that lymphatic vessels (LVs) may serve as a new target organ for the treatment of chronic inflammatory arthritis [42].

Numerous clinical and mouse studies have reported that inflammation is associated with reduced lymphatic drainage from adjacent tissues. Inflammation-induced lymphatic dysfunction may involve many mechanisms. Recently, we demonstrated that TNF promotes lymphatic endothelial cells (LECs) to produce nitric oxide (NO) as a result of iNOS over-expression, which directly inhibits functional gene expression by lymphatic muscle cells (LMCs) [15]. Alternating contraction and relaxation of LMCs propel lymph to draining lymph nodes and eventually to the venous circulation [4, 5]. However, the direct involvement of LMCs in RA-associated lymphatic dysfunction has not been well investigated. In the current study, we demonstrated for the first time that the progression of ankle arthritis in TNF-Tg mice coincides with the loss of LMC coverage on collecting LVs, and LMC degeneration/apoptosis. This can be restored by the herbal drug PNS. At the molecular level, PNS inhibits TNF-induced NO production by LECs and indirectly prevents the inhibitory effect of NO on LMCs. Our study highlights the importance of LMCs in controlling LV function in RA.

It is well known that newly formed LVs undergo intrinsic remodeling to mature into collecting vessels. This remodeling process includes covering the collecting vessels with LMCs and the formation of intraluminal valves [43, 44]. Smooth muscle cell coverage is, therefore, a marker of vessel maturation, and the reduced smooth muscle cell coverage represents the degenerative feature of collecting vessels [45, 46]. The data from our whole-mount staining revealed significantly reduced lymphatic vessels covered by LMCs in TNF-Tg mice with severe arthritis (Fig. 2). It can be deduced that the progression of inflammation causes the abnormal remodeling and degeneration of collecting LVs. This notion is further supported by our EM findings of the degenerative and apoptotic appearance of LMCs as well as adjacent LECs (Fig. 3). Thus, the lack of smooth muscle cells around the collecting vessels of old TNF-Tg mice is likely associated with cell apoptosis or detachment from LECs.

To explore the potential mechanisms for abnormal LMCs in TNF-Tg mice, we demonstrated that TNF decreases growth and induces apoptosis of LMCs in a time-dependent manner (Fig. 4). It also reduces the functional muscle gene expression of these cells. These results suggest a direct inhibitory effect of TNF on LMCs. LMCs can be exposed to TNF in vivo that is produced by infiltrated immune and inflammatory cells surrounding the outer collecting LVs [38, 39].

PNS has been used to treat patients with arthritis [19, 20]. The mechanisms of PNS are complex, including the inhibition of intracellular-free calcium concentration [47] and the stimulation of VEGF signals [48]. We reported that PNS promotes lymphangiogenesis in zebrafish and increases tube formation of LECs [24]. Our in vivo findings (Fig. 5) that PNS restores the draining function and structural integrity of collecting LVs in TNF-Tg mice further demonstrate the benefits of PNS in lymphatics. Interestingly, PNS does abolish the inhibitory effect of TNF on LMCs, but instead restores LMC function by inhibiting NO production by LECs (Fig. 6). PNS decreases the levels of nitric oxide and iNOS activity in lung tissue [23]. High concentrations of NO induce apoptosis of cancer cells [16], cardiomyocytes [17], and nerve cells [49, 50]. We found that iNOS inhibition prevents LMCs apoptosis induced by TNF-pretreated LECs (Fig. 6c). Taken together, our data suggest that at RA joints where TNF levels are high, TNF inhibits the function of LMCs via both direct and indirect mechanisms, leading to lymphatic dysfunction. PNS restores lymphatic vessel function by reducing TNF-mediated LMC apoptosis via inhibition of LEC NO production (Fig. 6f). In the current study, PNA treatment cannot rescue the gene expression of LMCs, whereas the expression of h1-cal or sMYH is recovered to a comparable level of the PBS treated group in LECs. We speculate that PNA may have a cell-specific effect, e.g., it affects LECs, but not LMCs. It is also possible that PNA regulates NO production, but not the expression of muscle genes. More studies are needed to elucidate the mechanisms of action of PNA on LMCs vs. LECs.

In order to verify the contribution of LMCs to the progression of arthritis, it is important to compare the phenotype observed in LMCs isolated from both WT and TNF-Tg mice and know if these LMCs from TNF-Tg mice play a role in inflammation. For instance, are they able to secrete inflammatory mediators like other muscular cells, and do they activate the lymphatic endothelial cells? Since there are no markers specific for LMCs, it is very difficult technically to distinguish LMCs from vascular muscle cells. To investigate the potential contribution of LMCs to RA pathogenesis, we recently used the lineage tracing approach and reported that LMCs covering popliteal lymphatic vessels, the joint-draining lymphatic vessels, are Pax7−/MyoD−/Prrx1+/NG2−, which differ from skeletal and vascular muscle cells (Kenney et al. Lineage tracing reveals evidence of a popliteal lymphatic muscle progenitor cell that is distinct from skeletal and vascular muscle progenitors. Scientific Reports. 10:18088, 2020). With this new set of LMC markers, we are currently performing single-cell sequencing on LMCs (Prrx1+/NG2−) isolated from popliteal lymphatic vessels of TNF-Tg mice and WT littermates. We anticipate that LMCs from TNF-Tg mice will express a different gene profile when compared to WT LMCs, which will help us to investigate the molecular changes in LMCs under inflammation and vice versa.

Our study has several limitations. First, joints contain many cell types. We do not have evidence to indicate a direct effect of PNS on LECs or other cell types within the joint. We might be able to use a targeted approach by using nanoparticle encapsulating PNS with anti-PDPN or anti-LYVE-1 antibodies to deliver PNS to LECs. Second, we only examined 1 regimen for PNS treatment, e.g., once everyday for 3 months administration on 3-month-old TNF-Tg mice. It is possible that other regimens may have better effects, such as giving the drug at an earlier stage or decreasing frequency. Finally, we used a mouse model of inflammatory arthritis. Whether PNS has an effect on other forms of inflammatory arthritis, such as collagen-induced arthritis, requires additional study.

## Conclusions

In summary, using TNF-Tg mice as a model of chronic inflammatory arthritis, we demonstrated a global structural change of LVs that drain hind limbs during the progression of ankle arthritis. Collecting LVs had a marked reduction in the LMC coverage, and LMCs showed a degenerative and apoptotic appearance. TNF caused LMC death directly and indirectly via the stimulation of NO production by LECs. The herbal drug PNS attenuated joint tissue damage, restored LMC coverage on collecting LVs, and improved lymphatic draining function. PNS did not directly affect the impact of TNF on LMCs but instead prevented TNF-induced NO production by LECs. Therefore, improving LMC function may represent a novel strategy and also serve as a new mechanism of action for drugs that have been used to treat patients with inflammatory arthritis.

## Supplementary Information

**Additional file 1: Figure S1.** Location of ankle tissues that are used for whole-mount immunofluorescence staining. Images of paws were from a WT mouse. Based on our preliminary findings, we used the ventral portion (A) for staining capillary LVs and the dorsal portion (B) for staining mature LVs. **Figure S2.** Changes in morphology and expression of smooth muscle myosin heavy chain 2 in different passages of lymphatic muscle cells. Primary rat lymphatic muscle cells were cultured. Cells were harvested at different passages. (A) Images of bright fields show morphologic changes. (B) Expression of smooth muscle myosin heavy chain 2 (SMYH2) by Western blot analysis. **Figure S3.** IL-6 did not significantly affect the cell growth and apoptosis of LMCs. Lymphatic muscle cells were treated with 0, 2.2, 6, 20 ng/ml IL-6 for 1 to 7 days. (A) Cell growth was assessed by an MTT assay. Values are mean ± SD of 3 samples. (B) Cell apoptosis was determined by flow cytometry. **Figure S4.** PNS did not significantly affect the weight of TNF-Tg mice. Weight of 3-month-old TNF-Tg mice were treated with PNS or saline by gavage daily for 3 months. *n* = 7 mice/group, NS, *p* > 0.05 by student *t* test.

## Data Availability

All data are available from the corresponding authors upon reasonable request.

## Abbreviations

LV: Lymphatic vessel
LMCs: Lymphatic muscle cells
TNF-Tg: Tumor necrosis factor-transgenic transgenic
PNS: Panax notoginseng saponins
NO: Nitric oxide
LECs: Lymphatic endothelial cells
iNOS: Inducible nitric oxide synthase
RA: Rheumatoid arthritis
WT: Wild-type
NIR-ICG: Near-infrared indocyanine green
H&E: Hematoxylin and eosin
LYVE-1: Lymphatic vessel endothelial hyaluronan receptor
PDPN: Podoplanin
SMA: α-Smooth muscle actin
ROI: Region of interest
Ami: Aminoguanidine hemisulfate salt
sMYH: Smooth muscle myosin heavy chain
hi-Cal: H1-calponin
SMa: Smooth muscle actin
SPF: Specific pathogen-free
LPS: Lipopolysaccharides
EDTA: Ethylene diamine tetraacetic acid
PCR: Polymerase chain reaction
SDS-PAGE: SDS-polyacrylamide gel electrophoresis
pERK: Phosphorylation of extracellular regulated protein kinases
SD: Standard deviation
ANOVA: Analysis of variance
VEGFR-3: Vascular endothelial growth factor receptor
VEGF-C: Vascular endothelial growth factor
EM: Electron microscope
