# Supplementary material for: Lymphatic muscle cells contribute to dysfunction of the synovial lymphatic system in inflammatory arthritis in mice
Source: Arthritis Res Ther. 2021 Feb 19;23:58. doi: 10.1186/s13075-021-02438-6 (PMC7893868; doi:10.1186/s13075-021-02438-6)
Supplement: Supplementary file 1 — Additional file 1: Figure S1. Location of ankle tissues that are used for whole-mount immunofluorescence staining. Images of paws were from a WT mouse. Based on our preliminary findings, we used the ventral portion (A) for staining capillary LVs and the dorsal portion (B) for staining mature LVs. Figure S2. Changes in morphology and expression of smooth muscle myosin heavy chain 2 in different passages of lymphatic muscle cells. Primary rat lymphatic muscle cells were cultured. Cells were harvested at different passages. (A) Images of bright fields show morphologic changes. (B) Expression of smooth muscle myosin heavy chain 2 (SMYH2) by Western blot analysis. Figure S3. IL-6 did not significantly affect the cell growth and apoptosis of LMCs. Lymphatic muscle cells were treated with 0, 2.2, 6, 20 ng/ml IL-6 for 1 to 7 days. (A) Cell growth was assessed by an MTT assay. Values are mean ± SD of 3 samples. (B) Cell apoptosis was determined by flow cytometry. Figure S4. PNS did not significantly affect the weight of TNF-Tg mice. Weight of 3-month-old TNF-Tg mice were treated with PNS or saline by gavage daily for 3 months. n = 7 mice/group, NS, p > 0.05 by student t test. [file 13075_2021_2438_MOESM1_ESM.pptx]

## Slide 1
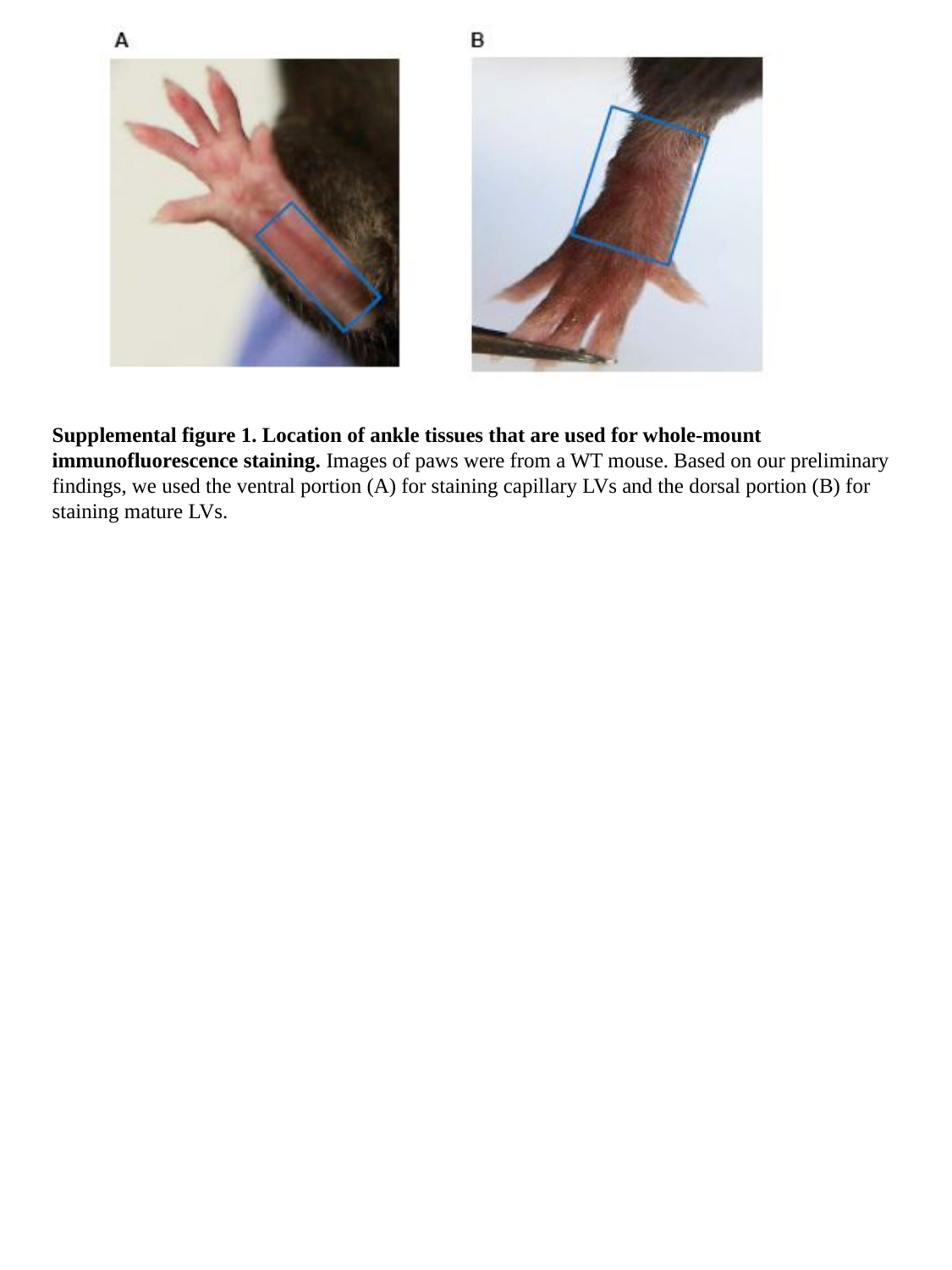

Supplemental figure 1. Location of ankle tissues that are used for whole-mount immunofluorescence staining. Images of paws were from a WT mouse. Based on our preliminary findings, we used the ventral portion (A) for staining capillary LVs and the dorsal portion (B) for staining mature LVs.

## Slide 2
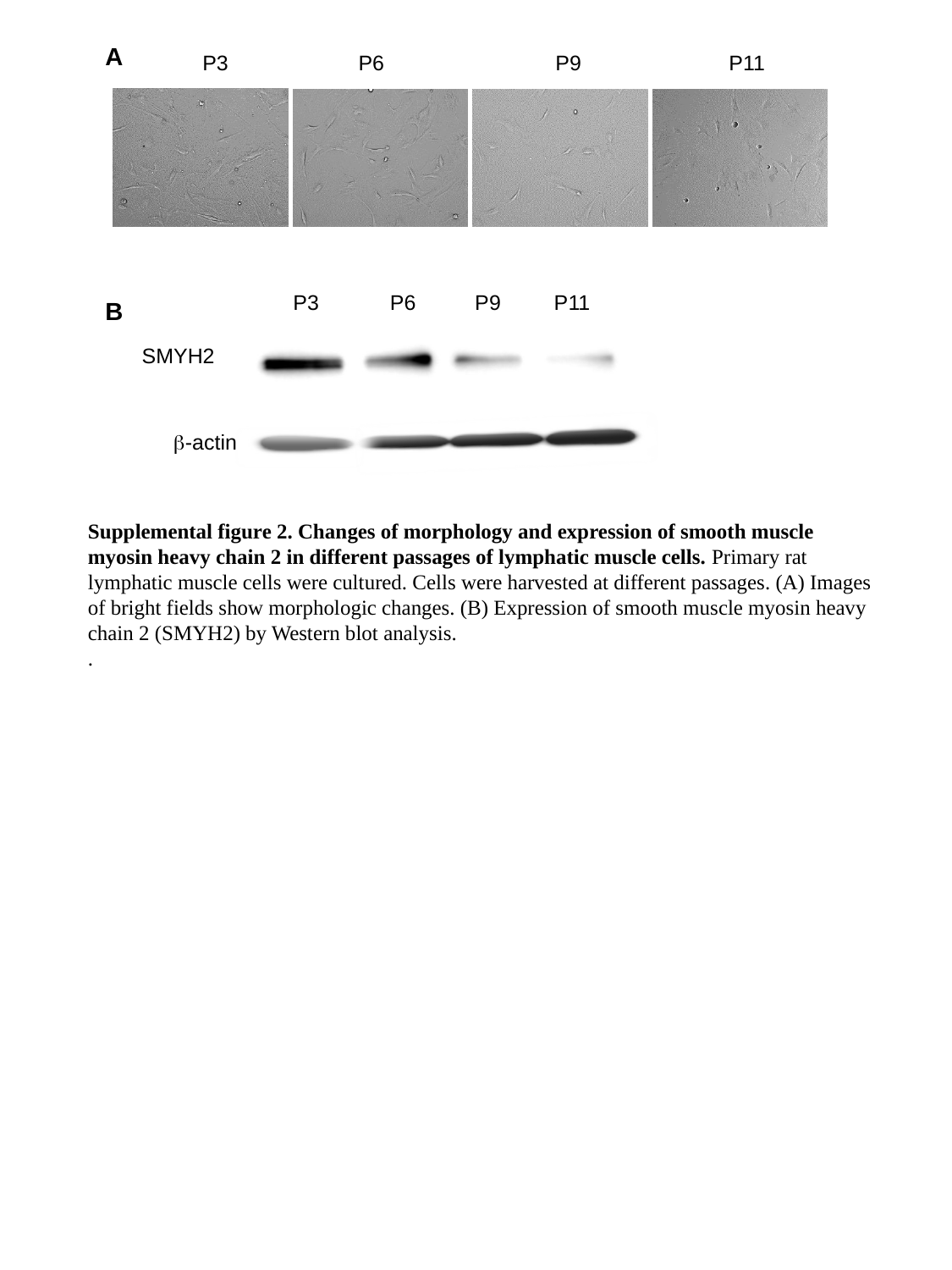

A
 P3 P6 P9 P11
 P3 P6 P9 P11
SMYH2
 b-actin
B
Supplemental figure 2. Changes of morphology and expression of smooth muscle myosin heavy chain 2 in different passages of lymphatic muscle cells. Primary rat lymphatic muscle cells were cultured. Cells were harvested at different passages. (A) Images of bright fields show morphologic changes. (B) Expression of smooth muscle myosin heavy chain 2 (SMYH2) by Western blot analysis.
.

## Slide 3
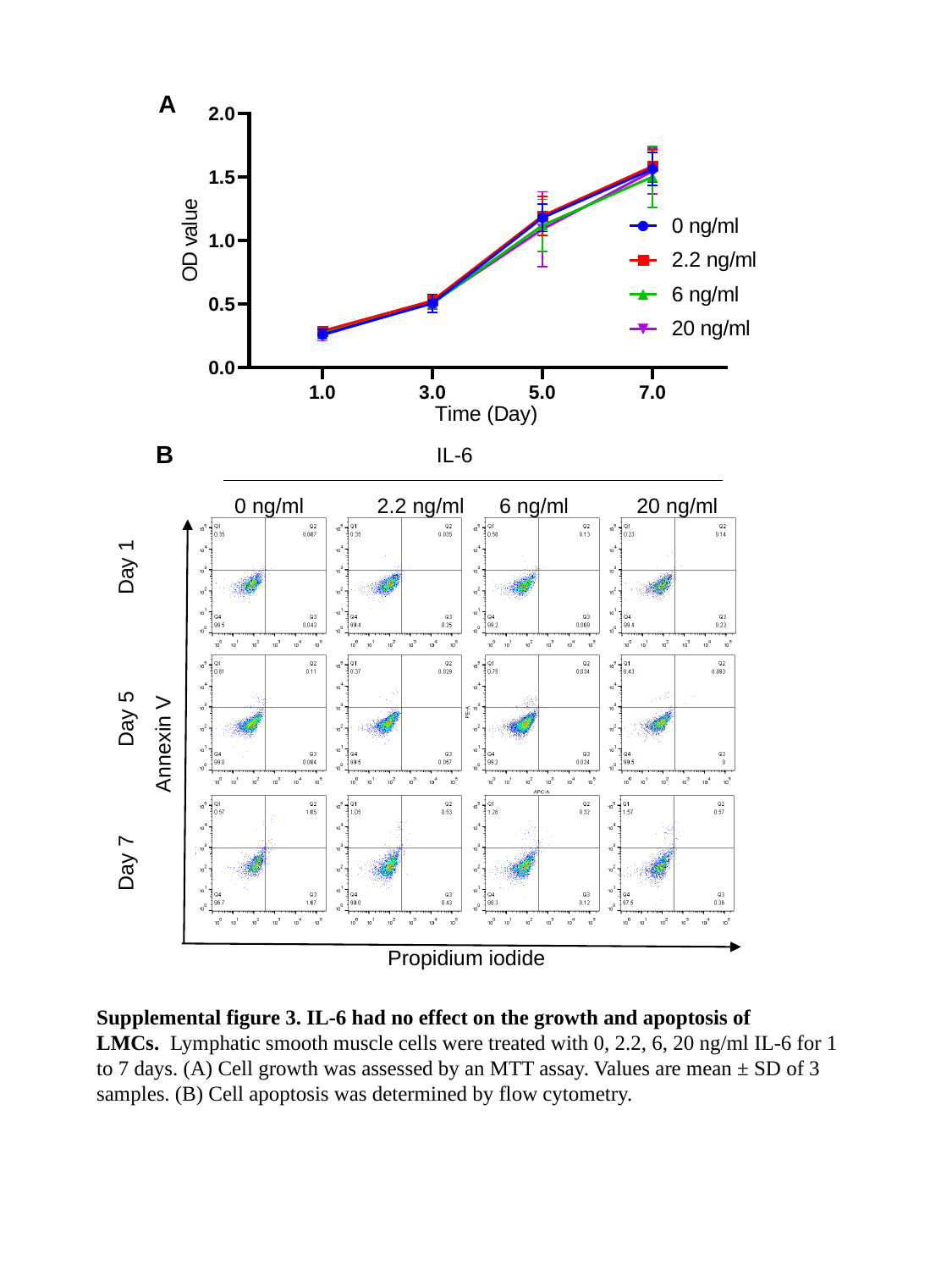

A
B
IL-6
0 ng/ml
2.2 ng/ml
6 ng/ml
20 ng/ml
Day 1
Day 5
Annexin V
Day 7
Propidium iodide
Supplemental figure 3. IL-6 had no effect on the growth and apoptosis of LMCs.  Lymphatic smooth muscle cells were treated with 0, 2.2, 6, 20 ng/ml IL-6 for 1 to 7 days. (A) Cell growth was assessed by an MTT assay. Values are mean ± SD of 3 samples. (B) Cell apoptosis was determined by flow cytometry.

## Slide 4
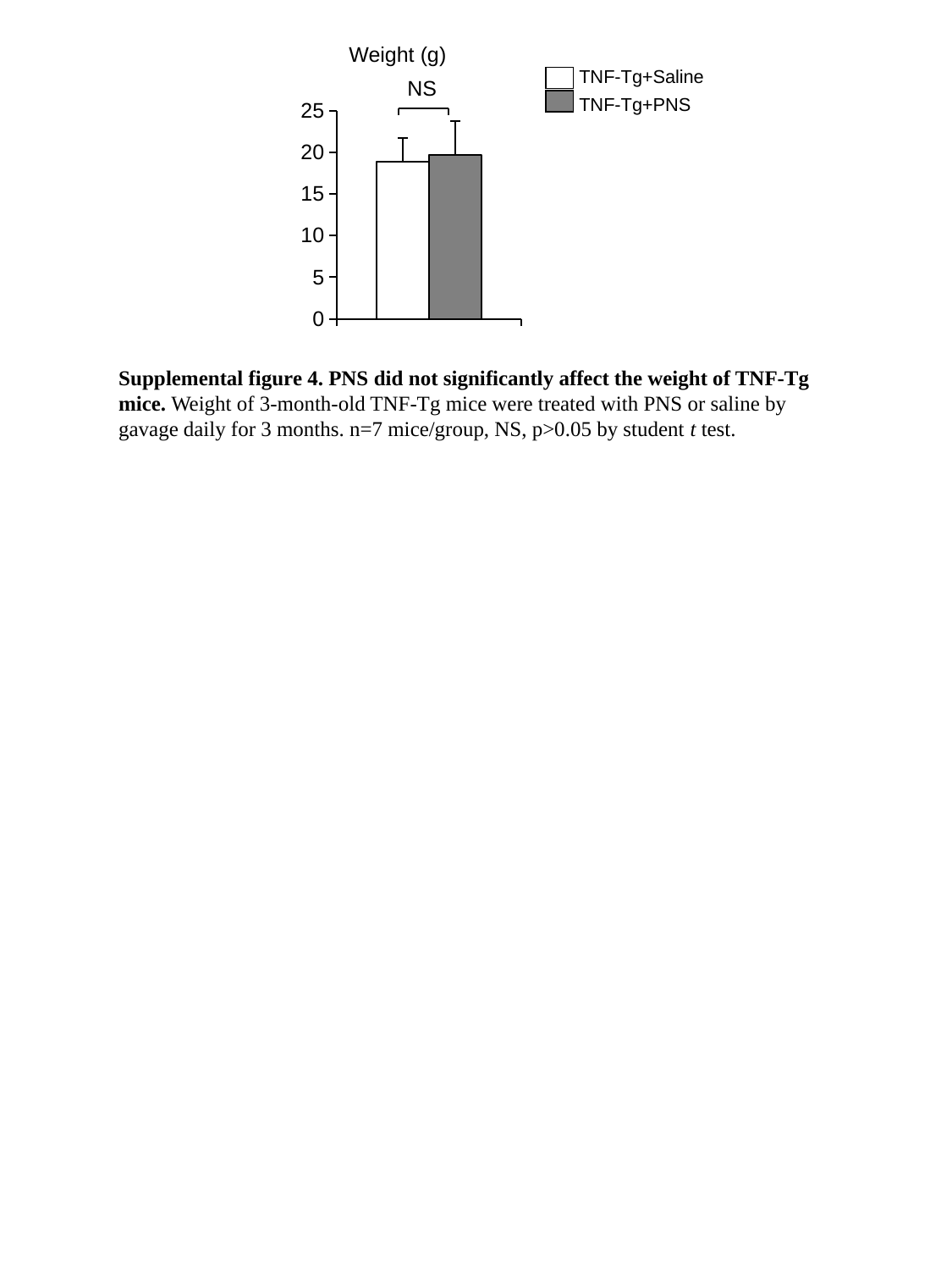

Weight (g)
TNF-Tg+Saline
TNF-Tg+PNS
NS
### Chart
| Category | Saline | PNS |
|---|---|---|Supplemental figure 4. PNS did not significantly affect the weight of TNF-Tg mice. Weight of 3-month-old TNF-Tg mice were treated with PNS or saline by gavage daily for 3 months. n=7 mice/group, NS, p>0.05 by student t test.
